# Comparison of Bacterial Risk in Cryo AHF and Pathogen Reduced Cryoprecipitated Fibrinogen Complex

**DOI:** 10.3390/pathogens11070744

**Published:** 2022-06-30

**Authors:** Thea Lu, Pallavi Nahata, Aja Johnson, Nadia Keltner, Lindsay Peters, Melissa McCormack, Bianca Muñoz, Mary Krath, Elan Weiner, Peter Bringmann

**Affiliations:** Cerus Corporation, Concord, CA 94520, USA; pnahata@cerus.com (P.N.); ajohnson@cerus.com (A.J.); nkeltner@cerus.com (N.K.); lpeters@cerus.com (L.P.); mmccormack@cerus.com (M.M.); bmunoz@cerus.com (B.M.); mkrath@cerus.com (M.K.); eweiner@cerus.com (E.W.); pbringmann@cerus.com (P.B.)

**Keywords:** fibrinogen, cryo AHF, cryoprecipitated antihaemophilic factor, INTERCEPT, hemorrhage, trauma, bacterial risk, massive transfusion protocol MTP, patient blood management PBM, sepsis

## Abstract

Until November 2020, cryoprecipitated antihaemophilic factor (cryo AHF) was the only United States Food and Drug Administration (FDA)-approved fibrinogen source to treat acquired bleeding. The post-thaw shelf life of cryo AHF is limited, in part, by infectious disease risk. Concerns over product wastage demand that cryo AHF is thawed as needed, with thawing times delaying the treatment of coagulopathic patients. In November 2020, the FDA approved Pathogen Reduced Cryoprecipitated Fibrinogen Complex for the treatment and control of bleeding, including massive hemorrhage, associated with fibrinogen deficiency. Pathogen Reduced Cryoprecipitated Fibrinogen Complex (also known as INTERCEPT^®^ Fibrinogen Complex, IFC) has a five-day post-thaw room-temperature shelf life. Unlike cryo AHF, manufacturing of IFC includes broad spectrum pathogen reduction (Amotosalen + UVA), enabling this extended post-thaw shelf life. In this study, we investigated the risk of bacterial contamination persisting through the cryoprecipitation manufacturing process of cryo AHF and IFC. Experiments were performed which included spiking plasma with bacteria prior to cryoprecipitation, and bacterial survival was analyzed at each step of the manufacturing process. The results show that while bacteria survive cryo AHF manufacturing, IFC remains sterile through to the end of shelf life and beyond. IFC, with a five-day post-thaw shelf life, allows the product to be sustainably thawed in advance, facilitating immediate access to concentrated fibrinogen and other key clotting factors for the treatment of bleeding patients.

## 1. Introduction

Hemorrhage rapidly depletes fibrinogen and other key clotting factors [1]. Fibrinogen levels lower than ~200 mg/dL are an independent risk factor for severe hemorrhage [2,3,4]. Replenishing these components is important for forming stable clots and hemostasis. Recent studies have demonstrated the benefits of early replenishment of fibrinogen, namely faster bleeding control resulting in improved outcomes, among pediatric [5], trauma [6], and post-partum hemorrhage [7] patients, making a strong argument to address these deficiencies earlier. The positive impact such care has on patient blood management and overall blood utilization [8], at a time when blood supplies are strained, furthers the need for early administration of fibrinogen and key clotting factors.

As fibrinogen concentrates are limited in their constituency and are not FDA-approved for treatment of acquired bleeding [9,10], cryoprecipitated antihaemophilic factor (cryo AHF) was until recently the only product available to wholistically replace fibrinogen plus multiple depleted clotting proteins [11]. Patients who received cryo AHF during massive bleeding events experienced reduction in blood product utilization, morbidity, and mortality [6,8,12,13,14,15]. Research has led to the consensus that early transfusion of cryo AHF in hemorrhage treatment is favorable to patients [13,14,16]. Time to cryo AHF transfusion is frequently measured in hours after patient arrival at emergency services or MTP activation. PROMMPT, a large trauma trial, showed that the median time from patient admission to receiving the first cryoprecipitate unit was 2.7 h (IQR: 1.7–4.4 h) [17]. McQuilten et al. published a variety of indications including trauma, CV surgery, and obstetric hemorrhage, and showed similar delays in access to cryo AHF, with an overall median of 2.5 h.

Several challenges present when institutions attempt to implement earlier cryo AHF administration, largely associated with the product’s limited post-thaw shelf life of 4–6 h, resulting in an inability to prepare the product in advance of requirement without meaningful logistical challenges and wastage [6,15,16] that govern the timing of product availability. Two recent studies—Curry et al. and Green et al.—focused on the feasibility of transfusing cryo AHF into actively bleeding patients within 90 min and experienced several obstacles in achieving the target [6,7].

Improving time until cryo AHF administration is facilitated by the extension of product post-thaw shelf life beyond the current 4–6 h, which has not been possible due in part to high risk of infectious disease; specifically, bacterial contamination. While fibrinogen and other key clotting factors remain stable for up to 10 days post-thaw, bacterial colonization and breakthrough infections begin as early as 4 h post-thaw [16,18,19].

Pathogen Reduced Cryoprecipitated Fibrinogen Complex (IFC), prepared from plasma treated with the INTERCEPT Blood System for Plasma and then processed with the INTERCEPT^®^ Blood System for Cryoprecipitation [20], is FDA-approved for the treatment and control of bleeding, including massive hemorrhage, associated with fibrinogen deficiency.

Both cryo AHF and IFC are cryoprecipitate products, manufactured by the cryoprecipitation of volunteer human plasma. Unlike cryo AHF, IFC undergoes treatment by the INTERCEPT Blood System (Amotosalen + UVA light) [21], which provides broad spectrum pathogen reduction and has a five-day post-thaw room-temperature shelf life. IFC is the only concentrated source of fibrinogen and other key clotting factors that can be thawed in advance, providing immediate access for bleeding patients without high wastage risk.

This paper will compare the risk of bacterial contamination between cryo AHF and IFC, to further address the logistical challenges associated with early fibrinogen administration to bleeding patients.

## 2. Results

### 2.1. Bacterial Survival during and after the Manufacture of Cryoprecipitated Products

**Figure 1:**
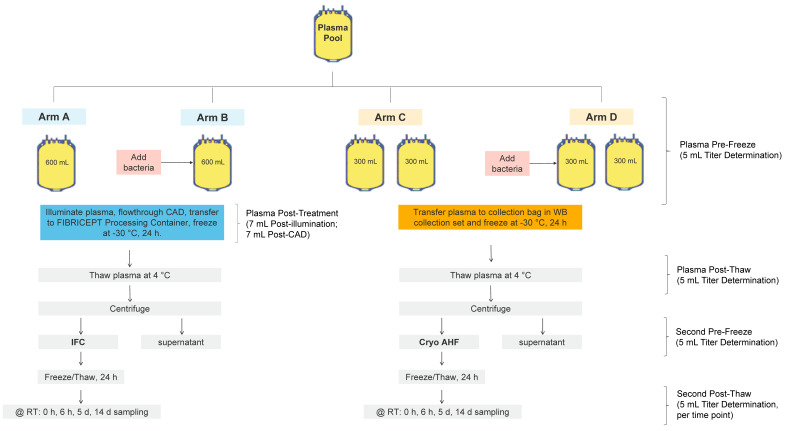
Study design for the assessment of bacterial survival during and after the process of manufacturing IFC and cryo AHF.

*Staphylococcus epidermidis*, *Pseudomonas aeruginosa*, and *Serratia marcescens* were used in this study because they have been observed in transfusion-transmitted infections and have been identified in contaminated blood products [22,23,24]. Additionally, these bacteria are able to survive in cryoprecipitate at room temperature [16,18] Bacterial survival of *S. epidermidis*, *P. aeruginosa*, and *S. marcescens*, throughout and after the process of manufacturing cryo AHF and IFC (Figure 1), showed retention of infectious disease risk for products that had not undergone pathogen reduction with the INTERCEPT Blood System.

### 2.2. Bacterial Survival in Plasma under Extended Hold Conditions

Prior to the cryoprecipitation manufacturing process, for cryo AHF or IFC, plasma components are typically held at either 4 °C or room temperature (approximately 20–24 °C) [25]. Some bacteria have been observed to grow to high titers in plasma even after one day of storage at room temperature [23,26], therefore bacterial titer was assessed for *S. marcescens* after the hold time and post-amotosalen/UVA treatment (Table 1). *S. marcescens* was chosen due to its ability to grow in a wide temperature range and survive in cryoprecipitate [16]. After holding at 4 °C, *S. marcescens* decreased from 74.0 ± 15.6 cfu/unit to 17 ± 13 cfu/unit. However, *S. marcescens* grew under room-temperature conditions (25 °C) to 5.6 log cfu/unit. This assessment indicates bacterial survival in plasma if the plasma was not further processed into cryoprecipitate. After the contaminated units were held in these conditions, both units were treated with amotosalen/UVA, and the entire unit was assessed for bacterial contamination and titer. No viable bacteria were detected in either condition after INTERCEPT pathogen reduction treatment.

### 2.3. High Inoculum Bacterial Survival during and after the Manufacture of Cryo AHF and IFC

Bacterial survival during the manufacture of cryo AHF and IFC and storage for up to five days at room temperature (approximately 20–24 °C) was assessed using a high inoculum of two bacteria frequently found in transfusion-transmitted infections [27,28,29]. *Pseudomonas aeruginosa* and *Staphylococcus epidermidis* were inoculated at 6.8 log cfu/mL and 6.7 log cfu/mL respectively (Table 2 and Table 3). *P. aeruginosa* and *S. epidermidis* were not detectable immediately after amotosalen/UVA treatment. No bacteria were detected during other steps in the cryoprecipitation manufacturing process, nor at five days post-storage of IFC. Without amotosalen/UVA treatment, both bacterial strains were detected after the initial freeze–thaw cycle, and in the subsequent processing steps, indicating bacterial survival during the manufacturing process. Additional growth of *P. aeruginosa* to 9.1 log cfu/mL and *S. epidermidis* to 7.3 log cfu/mL was observed after five days storage at room temperature in the cryo AHF that had not been pathogen reduced.

### 2.4. Low Inoculum Bacterial Survival during and after the Manufacture of Cryo AHF and IFC

*Serratia* species are potential contaminants and biofilm formers and have been observed to survive in low temperatures at a low inoculum [24]. Both low inocula and biofilms may initially remain undetectable but proliferate under the conditions found in stored blood components. Bacterial survival during the manufacture of cryo AHF and IFC and storage up to 14 days at room temperature (approximately 20–24 °C) was assessed using an inoculum of *S. marcescens* of approximately 1000 cfu/unit (Table 4). *S. marcescens* was completely inactivated after amotosalen/UVA treatment and no bacteria were detected during other steps in the cryoprecipitation process, or at 14 days post-storage. Without amotosalen/UVA treatment (Figure 1, Arm D), bacteria were detected after the initial freeze–thaw cycle, and the subsequent processing steps, indicating bacterial survival during the cryo AHF manufacturing process. Additional growth of *S. marcescens* to 9.4 log cfu/mL was observed after 14 days storage at room temperature.

## 3. Discussion

Potential bacterial contamination of cryo AHF at levels that represent a significant risk to the health of a transfusion recipient prevents the extension of the shelf-life of cryo AHF beyond 4 to 6 h, despite the demonstrated stability of fibrinogen and other coagulation factors [16,18,19]. This study investigated the survival of three transfusion-relevant bacterial species at concentrations mimicking initial contamination conditions during the cryoprecipitation manufacturing process. Bacteria were spiked into plasma, which was then processed into cryo AHF or a paired IFC component following Amotosalen/UVA treatment. Bacterial survival was demonstrated during and after the manufacture of cryo AHF, specifically for *S. epidermidis*, *P. aeruginosa*, and *S. marcescens*. During the extended hold conditions that plasma may experience prior to being manufactured into cryo AHF, bacteria can reach very high titers (>5 log cfu/mL) if held at room temperature for 18 h (Table 1). However, two freeze–thaw steps during cryo AHF manufacturing are not sufficient to eliminate all bacteria in plasma even if present at significantly lower titers (~1000 cfu/unit) (Table 4).

Previous studies indicated that plasma can be contaminated with bacteria after the separation of whole blood into components, with the plasma itself having no bactericidal effect [30]. Wagner et al. showed that when spiked into whole blood to manufacture cryo AHF, bacteria survived the processing with six out of the nine organisms still being detectable after the cryo AHF production process [16]. Additionally, after spiking cryo AHF, there was positive detection of all nine bacteria after five days of storage at room temperature [16]. Ramirez-Arcos et al. demonstrated that cryo AHF stored at room temperature is a good substrate for bacterial growth, with three of the four bacteria tested showing proliferation to 1000-fold levels of increase [18]. Cryo AHF that had been spiked with 2 to 3 log cfu/mL bacteria was stored at 20–24 °C for up to 24 h, where *Pseudomonas aeruginosa*, *Pseudomonas putida*, and *Serratia liquefaciens* grew 4 to 5 log cfu/mL [18]. The current study corroborates these previous results and further extends our knowledge by providing bacterial survival titers during and after cryo AHF production.

Bacterial proliferation begins as early as 4 h post-thaw [16,18,19]. Low levels of bacterial contamination in plasma may potentially occur through processes such as collection from donors with subclinical bloodstream infections [31] or microbial skin infections and would retain infectious risk through the cryo AHF manufacturing process (Table 2). Even at the 6-h time point, over 100 cfu/unit were observed (Table 4). Beyond 6 h, bacteria proliferated to titers that reached up to 9 log cfu/mL (Table 4). Importantly, plasma that was spiked but underwent amotosalen/UVA treatment showed no breakthrough infectious capacity out to 14 days post-thaw in IFC in both high- and low-level contamination studies (Table 2, Table 3 and Table 4). The survival of bacteria through the cryo AHF production process and the proliferation of these organisms during storage without further mitigation such as amotosalen/UVA treatment or other pathogen reduction technologies [32,33] illustrates the risk of bacterial contamination in cryo AHF and that of transmission of these pathogens to transfusion recipients [16,18]. The infectious disease risk is not limited to bacteria, with case reports of fatal transfusion transmission of viruses, such as West Nile virus, linked to the transfusion of a single cryo AHF unit [19].

Early access to fibrinogen and other key clotting factors during hemorrhage treatment necessitates a product that can be stored ready for transfusion. Improving access to a cryoprecipitated source of fibrinogen and other key clotting factors requires extended room-temperature shelf life. INTERCEPT treatment of plasma does not significantly impact coagulation factors or the function of cryo [34,35]. As shown here, IFC, with a five-day post-thaw shelf life, allows this improved access without high wastage risk, and provides the additional benefit of reducing the risk of transfusion transmitted infections from not only bacteria, but also viruses and parasites [20,21].

## 4. Materials and Methods

### 4.1. Strains and Growth Conditions

The bacterial strains were provided by the California Department of Health Services (Richmond, California, USA) and the Paul Ehrlich Institute (Langen, Germany). *Staphylococcus epidermidis* CDHS 85A-2460, *Pseudomonas aeruginosa* CDHS 91A-5818, and *Serratia marcescens* PEI-B-P-56 were used in the freeze–thaw assays and pathogen inactivation assays. All strains were maintained in bead stocks at −80 °C in Luria–Bertani broth (LB; EMD Millipore, Burlington, MA, USA) containing 10% dimethyl sulphoxide (DMSO; MilliporeSigma, St. Louis, MO, USA). For aerobic cultures, a sterile loop was used to transfer one bead of the frozen bacterial cultures into 25 mL LB broth in a 250 mL flask. The cultures were incubated with agitation at 37 °C for 20–27 h.

### 4.2. Bacterial Titer Determination

The procedure used to enumerate bacteria was as follows: Each bacterial sample to be titered was either plated neat or serially diluted 10-fold in blood bank saline (BBS; Thermo Fisher Scientific, Waltham, MA, USA), enumerated by direct plating of 100 µL of each dilution onto three LB agar plates, and incubated at 37 °C for 24–48 h. The titer of each sample was calculated by determining the total number of colonies per countable dilution. Dilutions with an average of 25 to 250 colonies were used for titer determination. If the average colony counts in samples fell below this range, all counts were used for the titer determination. Following the scoring, the observed bacterial titers of each sample were calculated in cfu/mL using the following formula:

Observed titer = (Total # colonies) ÷ ((Dilution) × (Total Volume)) = cfu/mL

### 4.3. Freeze–Thaw Assay

An overnight culture of bacteria was diluted 10-fold into either blood bank saline (BBS) or human plasma and then sampled to determine pre-freeze titer. The BBS and plasma with bacteria were then frozen for approximately 1 h at −80 °C (*S. epidermidis*, *P. aeruginosa*) or 24 h at −30 °C (*S. marcescens*) before being thawed at 37 °C. These samples were then plated to determine post-freeze titers.

### 4.4. Bacterial Survival in Plasma under Extended Hold Conditions Assay

Plasma components of 600 mL each were held at either 4 °C or room temperature (approximately 20–24 °C as defined by AABB standards [25]) prior to processing. To determine the growth of bacteria in plasma during these hold conditions, a plasma pool of 1200 mL was split into identical units of 600 mL each and 10–100 cfu of *S. marcescens* inoculated into each plasma unit. One arm was held for 18 h at 25 °C to represent the upper regulated room temperature threshold [36], and the second arm was held for 22 h at 4 °C (Table 1).

### 4.5. Preparation of Plasma, Amotosalen/UVA Treatment, and IFC or Cryo AHF Production

For each replicate, thawed apheresis plasma units clear of precipitates were pooled to reach a target volume of approximately 2500 mL (Figure 1). Next, the pool was split into four arms. Two INTERCEPT treatment arms had a target volume of approximately 600 mL each. The two cryo AHF arms were further subdivided into two arms, for a total of four units with a target volume of 300 mL each. As shown in Figure 1, a pool of plasma was split into two 600 mL units (arms A and B) and four 300 mL units (arms C and D). Arms B and D were inoculated with bacteria from overnight cultures diluted to obtain a target input inoculum (Table 2, Table 3 and Table 4). A 5 mL sample was removed from each unit for titer determination. Arms A and C served as the negative controls with no bacteria inoculated. Arms C and D were transferred to a collection bag and frozen at −30 °C for at least 24 h. Arms A and B were treated using the INTERCEPT Plasma Processing Set according to the instructions for use. The plasma unit was connected to an amotosalen pouch containing 15 mL of 6 mM amotosalen in 0.924% NaCl. The plasma flowed through the pouch into the illumination container, and plasma and amotosalen were mixed. Each plasma unit was then illuminated with UVA light at 6.4 J/cm^2^. After illumination was complete, a 7 mL post-illumination sample was removed from each arm to determine titer and to confirm treatment by HPLC. The plasma was then passed through the compound adsorption device (CAD) to remove residual amotosalen and free photoproducts. A 7 mL post-CAD sample was removed from each unit to determine titer and removal of amotosalen. After treatment, plasma was transferred to a new container of the INTERCEPT Blood System for Cryoprecipitation and stored at −30 °C for 24 h. After −30 °C storage, all units (arms A–D) were thawed at 4 °C for 24 h. At the end of the thaw process, 5 mL post-thaw samples were removed from all arms for titer determination and centrifuged to separate the precipitate. The thawed plasma was then centrifuged at 4 °C and 5857× *g* for 11 min to separate the precipitate, and the supernatant was removed. The precipitate pellets from the IFC units (arms A and B) were resuspended with 50 mL of the supernatant. For the cryo AHF (both units in arms C and D), approximately 25 mL of the supernatant was used to resuspend the cryo AHF pellet into the solution. The two sub-arms of the cryo AHF arm were then combined. A 5 mL pre-freeze sample was taken from each arm for titer determination. All units were then frozen at −30 °C for at least 24 h. At the end of the 24-h freeze, all units were thawed, and a 5 mL post-thaw cryoprecipitate (T = 0) was sampled for bacterial titer. All units were then held at 22–25 °C for 14 days with 12 mL samples taken at 6 h, five days, and 14 days for titer determination.

## 5. Conclusions

Patients with earlier access to cryoprecipitate during hemorrhage treatment experience a reduction in blood product usage, morbidity, and mortality [6,8,12,13,14,15]. Due to the potential risk of bacterial contamination, the short 4–6 h shelf-life of cryo AHF prevents thawed storage, necessitating the need to thaw the product for each patient, delaying fibrinogen replenishment. Bacteria can survive the cryoprecipitation process, which highlights the continued risk of bacterial contamination in the absence of additional mitigation strategies. The five-day, post-thaw shelf-life of IFC, which is manufactured from amotosalen/UVA-treated plasma, enables thawing in advance, without high wastage risk, providing clinicians with an immediate source of fibrinogen and other key clotting factors.

## Figures and Tables

**Table 1:** Survival of *Serratia marcescens* in plasma under extended hold conditions.

Hold Condition	Titer (cfu/unit)		
	Input	After Hold (Control)	Post-UVA (Test)
18 h at 25 °C	74.0 ± 15.6	3.7 × 10^5^ ± 2.7 × 10^5^	UD ^a^
22 h at 4 °C		17 ± 13	UD

^a^ UD = undetectable.

**Table 2:** Assessment of high inoculum *Pseudomonas aeruginosa* survival during and after the process of manufacturing IFC and cryo AHF for 5 days post-thaw storage.

Manufacturing Step		Titer (cfu/mL)	
		IFC	Cryo AHF
Plasma Pre-Freeze		6.3 × 10^6^	6.3 × 10^6^
Plasma Post-treatment		UD ^b^	-
Plasma Post-Thaw		UD	Too numerous to count ^a^
Second Pre-Freeze		UD	9.0 × 10^6^
Second Post-Thaw	0 h	UD	1.8 × 10^6^
	5 d	UD	1.3 × 10^9^

^a^ Further dilutions were not assessed for titer calculation. ^b^ UD = undetectable.

**Table 3:** Assessment of high inoculum *Staphylococcus epidermidis* survival during and after the process of manufacturing IFC and cryo AHF for 5 days post-thaw storage.

Manufacturing Step		Titer (cfu/mL)	
		IFC	Cryo AHF
Plasma Pre-Freeze		4.8 × 10^6^	4.8 × 10^6^
Plasma Post-treatment		UD ^b^	-
Plasma Post-Thaw		UD	Too numerous to count ^a^
Second Pre-Freeze		UD	Too numerous to count ^a^
Second Post-Thaw	0 h	UD	1.6 × 10^7^
	5 d	UD	1.8 × 10^7^

^a^ Further dilutions were not assessed for titer calculation. ^b^ UD = undetectable.

**Table 4:** Assessment of low inoculum *Serratia marcescens* survival during and after the process of manufacturing IFC and cryo AHF for 14 days post-thaw storage.

Manufacturing Step		Titer (cfu/unit)	
		IFC	Cryo AHF
Plasma Pre-Freeze		1261 ± 293	1273 ± 292
Plasma Post-treatment		UD ^b^	-
Plasma Post-Thaw		UD	998 ± 436
Second Pre-Freeze		UD	1224 ± 1026
Second Post-Thaw	0 h	UD	326 ± 55
	6 h	UD	131 ± 114
	5 d	UD	Too numerous to count ^a^
	14 d	UD	2.57 × 10^9^ ± 4.95 × 10^7^ cfu/mL

^a^ Further dilutions were not assessed for titer calculation. ^b^ UD = undetectable.

## Data Availability

The data presented in this study are available within the article.
